# Every doctor needs a wife: An old adage worth reexamining

**DOI:** 10.1007/s40037-019-0502-9

**Published:** 2019-03-26

**Authors:** Abigail Ford Winkel

**Affiliations:** New York University Langone Health, New York, USA

**Keywords:** Women in medicine, Healthcare workforce, Professionalism

## Abstract

Half of medical school graduates are women, but female doctors experience significant professional tensions. Low numbers of women in leadership roles, high burnout and attrition, and continued harassment suggest a culture that undermines the contributions of women. This manuscript explores research from sociology, business and medicine through a personal lens. Understanding the way gender influences the complex state of women in medicine suggests changes are needed in the architecture of the modern medical workforce. Individuals, mentors and organizations can make changes that would improve the way that the working environment cultivates a diverse workforce to reach its full potential.

*Looking up at the podium in a big auditorium in my first year of medical school, I first heard this expression from the dean of students, as she towered over us. The words didn’t fit. What did she mean by ‘every doctor needs a wife’? Over the fifteen years since then, I have begun to understand.*

My own journey to becoming a doctor, wife, and mother provided perspective on those words. The phrase *‘every doctor needs a wife*’ does not encapsulate the complexity facing women in medicine. As an obstetrician-gynaecologist and educator, I have learned from and worked with many women navigating this territory. Research published in the medicine, sociology and business literature suggests a seismic shift is underway. Exploring insights raised by this research through the lens of my own experiences leads to suggestions for how medicine can move forward.

## A rose by any other name

### The power behind words to define roles and expectations

*What is a ‘wife’ anyway? The word conjures up so many images. For me, the one that arises is my grandmother, wife to a veterinarian and mother of 11 children. She would host 40 people for holiday meals. She hummed as she vacuumed the house, which she seemed to do constantly. My cousins and I saw her as happy, full of pride in her family. She and my grandfather were teammates. Each had their distinct arena, their clear roles.*

The word ‘wife’ is shorthand for many things—household management, child-rearing, emotional support—roles my grandmother performed. While the expression may refer to the *role*, rather than the actual *person* of wife, it nonetheless calls up a tightly gendered image in our cultural memory. This idea influences women’s lives even as roles have changed. Feminism in the 1970s saw women taking control of their bodies and gaining admission into exclusive clubs [1]. But women have assimilated rather than changed the systems they entered [2]. Entrenched gender archetypes continue to reveal values and stereotypes.

## From quota to cohort

### The change in representation of women in medicine

Women have come a long way since the mid-19th century founding of all-women’s medical schools. Recently, equal numbers of women and men graduate medical school [2, 3]. But while the gender profile of doctors has evolved, medical training has not. The structure was designed in the 1960s for men with few household duties [4]. There are signs of tension in the careers of female doctors [5]. Women are more likely to leave academic medicine than men before becoming associate professors [6]. In 2014, women represented 38% of full-time medical school faculty, 21% of full professors, 15% of department chairs, and 16% of deans [7].

Why has the prominence of women physicians lagged behind? The phenomenon is seen in other fields. Women earn half of professional degrees and occupy managerial roles, but few reach the upper echelons of leadership [8]. Across industries, qualified women leaving the workforce is causing a ‘brain drain’ [9]. Sociologists give three explanations for this: women opt out of opportunities based on different values; women spend time on non-work priorities; and women face discriminatory barriers at work [10]. Each of these factors plays a role.

Gender stereotypes describe certain characteristics as masculine: aggressiveness, decisiveness, independence, and confidence. Other characteristics are seen as feminine: kindness, cooperation, concern for others [11]. Role congruity theory suggests that stereotypes prescribe norms—the ‘should’ and ‘should nots’—of how individuals behave [12]. Women may not aspire to roles that conflict with socially constructed values. A woman whose behaviour violates stereotype norms may be penalized in terms of social approval and performance evaluations [11, 13]. The result is fewer women come forward and those that do have a harder time.

## What women want

### The influence of gender on professional aspirations and achievements

*So what happens, then, when the doctor is a wife? I always wanted to be a doctor, but I remember the discrete moments of panic when I realized I would earn my medical degree before I married; when I learned about age-related declines in fertility. I feared that my professional choices would stand in the way of the life I had envisioned with a husband and children*.

*My mother has a PhD, and worked full-time as a single mother after my parents divorced. I didn’t see the professional trade-offs she faced, although she tells me they were there. What I saw was a mother who showed up to my field hockey games and stayed home when I was out of school sick. I did not think that I would have to choose between my professional aspirations and my hopes for a family.*

An exclusive focus on work is neither the goal nor the reality for professional women. Globally, successful women describe paths that account for both career and family [14]. Balancing these goals leads to trade-offs: women are more likely than men to take time off, work part-time or choose roles with fewer responsibilities—and lower salaries [9–11, 15, 16]. Female physicians spend more time doing domestic work, and are more likely to interrupt work or reduce their hours when they have children [17–19]. This pattern reflects society’s expectations as well as women’s own values. One study concluded, ‘for successful women in academic medicine, satisfaction *is* balance.’ [20]

*At the end of residency, I remember weighing the subspecialty fellowship I had wanted to pursue against the plans I was making in my home life. I was getting married. I was turning 30. I could not imagine spending another three years in training and put off my own journey to have children any longer. Within a few years, I had two healthy daughters and my professional growth continued. I have a work environment that values family, a supportive non-physician husband, a superhero nanny. But looking back, I have questions. Why didn’t I take a day off when I had a miscarriage? Why did I insist on breastfeeding my daughters over a year of pumping milk through tears on overnight calls?*

I count myself lucky. Research shows that academic productivity for female faculty is adversely affected by child-rearing [6]. If women take opportunities to work part-time, reduce hours, share a job or stop a tenure clock, they get less satisfying work and may be perceived less worthy for promotion [10, 21]. Flexibility policies intended to alleviate work-home conflict are associated with stigma of low professional commitment [6, 7, 9]. Women physicians around the world earn less than men [10, 20, 22, 23]. This gap widens with age, spreading dramatically in the 40–44 year age range [9, 13]. As an obstetrician, this timing seems chilling: at the same time careers accelerate, professional women may face consequences to fertility. Work-home tensions are cited as a barrier to women’s involvement in leadership roles, and a contributor to high levels of burnout and depression seen in women physicians [8, 24, 25].

Despite recognizing inhospitable practices, discriminations, and trade-offs, female physicians report satisfaction with their professional lives [10]. It is a paradox that so many women who remain engaged in their work report satisfaction while studies show high rates of burnout among female doctors. High rates of attrition from women leaving the medical workforce suggest that many do not feel similarly satisfied with the balance between work and family roles [24, 25].

## The clandestine power of gender in medicine

### The influence of gender on professional aspirations, expectations and evaluations

*Unlike women of earlier generations who were conscious of their trade-offs in breaking with gender roles, I grew up assuming all paths were open. I’m not alone here. Young, white women may not recognize discrimination unless it is grossly obvious* [12]. *But in retrospect, the role of gender is clearer.*

*From a very early age, I planned to be an orthopaedist. But as I worked in orthopaedic research labs and sports medicine clinics, I noticed two gendered roles available for women. I could retreat from my feminine side and assume a steely, aggressive pose. Or, I could giggle and tolerate being treated like a little girl. I didn’t think that gender had anything to do with it, per se. I just started to feel like I did not belong. In obstetrics and gynaecology, I saw strong, smart, capable women surgeons who were warm to their patients and colleagues. The job fit with the vision I had for myself as a doctor and a woman. I’m not the only one feeling this way; most of our residency applicants are women. But even in obstetrics and gynaecology, only 20% of department chairs are women* [26]

Gender plays a subtle role in modern medicine. Reports of frank harassment and obstruction are no longer the norm. But, it is naïve to pretend that these do not exist [16, 27]. A 2018 study of women physicians and their physician daughters revealed rampant, unchanged discrimination [13]. In interviews with women surgeons, each denied the role of gender in their own experience, but reported that discrimination was pervasive in their field [15]. Many medical students experience harassment [28]. Female doctors’ contributions may be undermined [4, 29]. It is not surprising that female physician leaders feel like cultural outsiders [8, 13].

Differences in both academic and clinical priorities for women influence their career trajectories. Valuing the broad impact of service, teaching and personal balance, women have fewer grants and publications than male peers [19, 20, 25]. It is rare for academic centres to consider the role that these other activities play in academic productivity [6]. No one doubts that there is value in this work: that value is just challenging to account for in measuring productivity. In clinical practice, women take more time with patients, display superior communication skills and practice more in line with guidelines. They also have lower productivity in terms of terms of hours worked and number of patients seen [16, 30]. A *New York Times *article asked ‘Should You Choose a Female Doctor?’ citing better outcomes for cardiac patients, surgical patients and Medicare recipients cared for by women physicians [16, 30–32]. These differences in outcomes are small, but these findings cast an important light on the question of ‘productivity.’ How could we better capture patient outcomes and satisfaction when measuring the quality of their physicians?

## Dress for the job you want, not the job you have

### Conflicting advice exists for how to achieve professional success

*I heard a lecture as a resident about how to succeed as a woman in academic medicine. The speaker gave advice based on what had worked in her life: hire someone to care for the household duties, hire someone else to care for the children, hire an assistant. The message was not so different from ‘Every Doctor Needs a Wife’, but she suggested that these duties could be outsourced. For many mentors and colleagues, this has been the secret to their success—a husband with a flexible job who can be the primary parent, a caregiver living in the home, a non-traditional family pattern that allowed someone else to be ‘wife’.*

*I wasn’t against getting help, but I wanted to participate in the intimate details of family life. The role models I truly admired had been able to be wives, to be mothers while also being great doctors. Delegating these roles felt like a loss. I wanted to make pancakes and walk my children to school. I wanted a job that would allow me to do these things myself, at least sometimes. I didn’t want to wear a suit to work every day. I felt as if I was disqualifying myself from paths forward because they didn’t ‘look’ like me.*

In academic medicine, faculty must be ‘individually successful contributors to their institution and society; committed professionals who work tirelessly for their patients, students, and the public; and role models of emotional, social and physical health. These expectations compete and no one achieves all of them all of the time’ [19]. Faculty achieving this standard have personal support to ‘devote the full force of their talents to these pursuits’ [19]. The culture favours the support that a traditional housewife might provide. Both young men and women question this narrow definition of success, favouring more flexibility and engagement in non-work roles [10, 19].

## Dr. Jekyll and Mrs. Hyde

### The importance of mentors to consider personal and professional goals

*In my own life, I see gains as well as losses in the trade-offs between work and home. My husband and daughters have a sweet private bedtime ritual when I am on call. When my kids are sick, our nanny cares for them as I am not the household manager my grandmother was. But I make the muffins when it is time for special snack at school. A mentor gave me treasured advice to think hard about when I need to be home, and when I don’t. Did I really need to give my kids a bath? Or was it enough for me to dive right into bedtime reading at the end of a long day?*

Role models who represent both a desirable career path and a desirable lifestyle are hard to find [25]. Strategies used by successful female physicians to navigate traditionally male roles can be isolating. Women silence themselves to gain acceptance or create ‘dual selves’ for different roles, and in doing so lose power to influence professional culture together [25, 33]. Individual women feel discouraged by role strain and concerns about their own competence [10, 34]. The ones who succeed continue to feel isolated within traditions that favour men [20, 33].

Mentors offer the potential to improve satisfaction by helping a mentee clarify values and navigate institutional barriers [20]. Sponsorship (publicly endorsing a mentee or offering an opportunity) may be more effective in promoting professional advancement than relational mentorship that focuses on professional style. Networking through professional societies may provide valuable, impartial supports [19]. Studies show that shared values between mentor and mentee are more important than shared gender [16]. Advice to female students to assume an assertive, masculine style is short-sighted [35]. In considering professional choices, one size does not fit all, and mentees should be challenged to question their sense that they do not ‘fit’ a particular role, as this may be the influence of stereotypes [19]. Given how important balancing activities in both personal and professional spheres seems to be for success, both work and home should be considered in mentoring. Mentees may need encouragement to navigate institutional barriers, or to consider whether stigma is preventing a mentee from taking advantage of existing flexibility policies. Mentors of female faculty will want to consider the complex landscape (Tab. 1) and encourage their mentee to reflect on their own goals (Tab. 2). The most engaging and productive career is the one that fits best with that individual’s overall life.

**Table 1:** A snapshot of the issues facing women physicians today

**Organizational policies, practices and culture**
Women represent half the medical workforce but are underrepresented in leadership roles [5].
Salary inequities exist between men and women doctors [5].
Flexibility policies (job-sharing, part-time work, stopping a tenure clock are rare and using them is associated with stigma) [26, 41].
Traditional metrics of productivity (clinical dollars, research grants) may underestimate women’s contributions (service, teaching, committees, mentoring, collaborative research) [11].
Women physicians approach their work differently, taking more time with patients and adhering to guidelines more. Some studies show improved patient outcomes for patients of women doctors [31, 33].
Work centric culture and unrealistic expectations on the part of colleagues may play a role in high rates of attrition among women physicians [10, 34].
Gender bias may result in discrimination, hostility and underestimating work done by women [11, 25, 29].
**Work-home conflict**
For women in academic medicine, ‘satisfaction is balance’ [20].
Real and perceived trade-offs exist for women between work and non-work life, parental and work roles, and delaying a family for the sake of not falling behind professionally [10].
Women physicians spend more time than their male peers doing domestic work [10].
Women more likely to work part-time or take time off for life events. There may not be an ‘on-ramp’ for doctors who take an ‘off-ramp’ [9].
Both men and women increasingly question narrow definitions of professional success, favouring more flexibility and engagement in non-work roles [10, 19].
**Role models and community**
More women enter academic medicine than men, but leave in disproportionate numbers before achieving the rank of associate professor [6].
Women balance goals at home and work and often have separate identities within these different spheres [34].
Women physicians report feeling isolated, invisible and marginalized [34].
Women leaders may cope with these challenges by self-silencing and creating micro-environments which do not lead to broader culture change [34].
Women leaders may be penalized in terms of social approval for professional competence when their behaviour violates gender expectations [11, 13].
There is a lack of role models and sponsors [9, 25].
Women of colour report being in a ‘double bind’ as outsiders [38].

**Table 2:** A guide for mentors

1. Effective mentors for women need not be women themselves.
2. Mentors should engage in both relational mentorship (coaching, advice) but also in concrete sponsorship (promoting mentee and identifying opportunities for advancement).
3. Help mentee to clarify values and goals in both personal and professional spheres. Encourage reflection on personal style and conflicts between approaches needed for success in these different arenas.
4. Discuss time management and look for ways to help balance work and life activities.
5. Challenge barriers that may reflect the mentee’s perception that they do not ‘fit’ a particular role. This may be the influence of stereotypes.
6. Look for professional development opportunities that exist through professional societies, especially those involving group work and networking with mentors [19, 41].
7. Identify institutional obstacles and discuss how these can be strategically navigated.
8. Encourage use of flexibility policies when they exist. Explore perceptions of stigma around making use of these options.
9. Coach women through career setbacks. Successfully navigating these leads to empowerment, resiliency and acceptance [20].
10. ‘Push out’ professional development opportunities to promising women rather than assuming these women will identify their own potential or suggest themselves for these roles unless they are overqualified.

## Looking ahead

*The other day, one of my most talented junior colleagues shared with me her concerns about her career. She was applying for a competitive position, and worried that she would be disqualified as a serious candidate because she had taken a short detour on a less demanding path during a critical time in her family. I encouraged her that she had gained valuable experience, and the short delay was insignificant in the timeline of her career. I could not guarantee that others would see her competence and potential the same way I did. But I did encourage her to pursue her path to leadership, knowing that she—and the diversity of experience she brings to the table—will bring us one step further in the right direction.*

Women make excellent doctors and leaders [31]. Across the globe, studies show that women leaders add value [8]. The number of female professionals is increasing, with disproportionate growth coming from underrepresented groups [9]. Minority women are in a ‘double bind’, and may be better equipped to identify the subtle biases operating in academic health centres [19, 33, 36]. Without diversity in leadership, the factors obstructing the progress of many gifted physicians will persist.

Statistics about disparities in rank, pay and representation do not tell the story of why doctors who do not fit traditional archetypes struggle over the arc of their careers [12]. Both practical adjustments and a cultural shift are needed to capture an individual’s contribution beyond traditional metrics [10, 19, 37]. Investing in flexibility policies, faculty development and mentoring will help cultivate a diverse workforce [9, 10].

There is an economic argument for investing in physician resilience, based on poor performance by burned out physicians [38, 39]. Addressing work-home conflict, exhaustion, and dissatisfaction will benefit both men and women. Professional dissatisfaction is increasing in younger male faculty [6, 40]. The work of academic medicine is inherently demanding. But there is no reason to hold fast to a system that fails to capture the full potential of its workforce.

## Call to arms

Does every doctor need a wife? No. But, she needs a village. She needs a revolution, and she needs a path to reach her full potential personally and professionally. Only by challenging the unexamined expectations for success will healthcare evolve to meet the needs of its doctors—and their patients.
